# Current Data on COVID-19 mRNA-Vaccine Safety during Pregnancy Might Be Subject to Selection Bias. Reply to Stroobandt, S.; Stroobandt, R. Data of the COVID-19 mRNA-Vaccine V-Safe Surveillance System and Pregnancy Registry Reveals Poor Embryonic and Second Trimester Fetal Survival Rate. Comment on “Stuckelberger et al. SARS-CoV-2 Vaccine Willingness among Pregnant and Breastfeeding Women during the First Pandemic Wave: A Cross-Sectional Study in Switzerland. *Viruses* 2021, *13*, 1199”

**DOI:** 10.3390/v13081546

**Published:** 2021-08-05

**Authors:** Sarah Stuckelberger, Guillaume Favre, Michael Ceulemans, Eva Gerbier, Valentine Lambelet, Milos Stojanov, Ursula Winterfeld, David Baud, Alice Panchaud, Léo Pomar

**Affiliations:** 1 Department Woman-Mother-Child, Lausanne University Hospital, University of Lausanne, 1011 Lausanne, Switzerland; Sarah.Stuckelberger@chuv.ch (S.S.); guillaume.favre@chuv.ch (G.F.); Eva.Gerbier@chuv.ch (E.G.); valentine.lambelet@gmail.com (V.L.); Milos.Stojanov@chuv.ch (M.S.); David.Baud@chuv.ch (D.B.); 2 Department of Pharmaceutical and Pharmacological Sciences, KU Leuven, 3000 Leuven, Belgium; michael.ceulemans@kuleuven.be; 3 Teratology Information Service, Pharmacovigilance Centre Lareb, 5237 MH ’s Hertogenbosch, The Netherlands; 4 Swiss Teratogen Information Service, Service de Pharmacologie Clinique, Lausanne University Hospital, University of Lausanne, 1011 Lausanne, Switzerland; Ursula.Winterfeld@chuv.ch; 5 Institute of Primary Health Care (BIHAM), University of Bern, 3012 Bern, Switzerland; alice.panchaud@chuv.ch; 6 Service of Pharmacy, Lausanne University Hospital, University of Lausanne, 1011 Lausanne, Switzerland; 7 School of Health Sciences (HESAV), University of Applied Sciences and Arts Western Switzerland, 1011 Lausanne, Switzerland

We would like to thank Stroobandt, S. and Stroobandt, R. for showing interest in our paper [1] and for sharing their concerns regarding COVID-19 vaccine safety in pregnant women [2]. Although not being the main focus of our article, it is a key component of COVID-19 vaccine acceptance in the pregnant woman population. 

Both authors are warning the readership of *Viruses* about the safety of COVID-19 mRNA vaccines, suggesting an interpretation error in the results of Shimabukuro et al.’s study which was considered as the first evidence of the safety of the COVID-19 vaccine in pregnancy [3]. Stroobandt, S. and Stroobandt, R.’s interpretation leads to an 82% risk of spontaneous abortion (104 spontaneous abortions for 127 participants exposed to the first dose of the COVID-19 vaccine during the first trimester and who have completed their pregnancy) instead of the 12.6% calculated by Shimabukuro and colleagues (104 spontaneous abortions for 807 participants with a pregnancy outcome at the time of analysis). As we totally disagree with their interpretation of these data, we would like to take the opportunity to respond to their letter.

Shimabukuro et al. decided to include in their analysis all women exposed to a COVID-19 mRNA vaccine during pregnancy and who had the chance to complete their pregnancy (i.e., live birth, spontaneous abortion, stillbirth, induced abortion and ectopic pregnancy). This conservative approach has led to a larger contribution to the study population of both patients with exposure occurring later in pregnancy and those with a short-term outcome, such as spontaneous abortion, as the available time to follow up was short. To our understanding, this study population might not have fairly represented the population at risk for spontaneous abortion and might have led to a selection of women ending with a spontaneous abortion. Spontaneous abortion incidence rates are sensitive to gestational age at enrollment as the risk decreases over gestation, with later enrollees carrying a lower risk, or no risk of the outcome as mentioned by Stroobandt, S. and Stroobandt, R. Thus, a dedicated analysis estimating the rate of spontaneous abortion by considering all women vaccinated during the first trimester who either had or were at risk of having a spontaneous abortion (i.e., beyond 20 weeks of amenorrhea at the time of the analysis), even if still with an ongoing pregnancy, would probably have provided a fairer estimate. With the information available in the paper by Shimabukuro et al., the spontaneous abortion risk would have probably been around 10% (104 spontaneous abortion for 1132 women vaccinated during the first trimester), which is even lower than the previously published estimate.

In our opinion, the debated data, along with other published data [4,5] show very reassuring results when evaluating the SARS-CoV-2 vaccination safety among pregnant women to date. This is of paramount importance as pregnant women are considered a vulnerable population for SARS-CoV-2 infection [6].
